# Immune Profiling Uncovers Memory T-Cell Responses with a Th17 Signature in Cancer Patients with Previous SARS-CoV-2 Infection Followed by mRNA Vaccination

**DOI:** 10.3390/cancers14184464

**Published:** 2022-09-14

**Authors:** Miriam Echaide, Ibone Labiano, Marina Delgado, Angela Fernández de Lascoiti, Patricia Ochoa, Maider Garnica, Pablo Ramos, Luisa Chocarro, Leticia Fernández, Hugo Arasanz, Ana Bocanegra, Ester Blanco, Sergio Piñeiro-Hermida, Pilar Morente, Ruth Vera, Maria Alsina, David Escors, Grazyna Kochan

**Affiliations:** 1 Oncoimmunology Group, Navarrabiomed, Fundación Miguel Servet-Hospital Universitario de Navarra-UPNA-IdISNA, Irunlarrea 3, 31008 Pamplona, Spain; 2 Oncobiona Group-Navarrabiomed-UPNA-IdiSNA, Irunlarrea 3, 31008 Pamplona, Spain; 3 Department of Medical Oncology, Hospital Universitario de Navarra-IdISNA, Irunlarrea 3, 31008 Pamplona, Spain

**Keywords:** SARS-CoV-2, oncology, vaccination, cancer

## Abstract

**Simple Summary:**

Cancer patients are considered a high-risk group for infectious diseases including COVID-19. The protective effects of vaccination are unclear in oncologic patients, as well as their duration. In this study antibody, T-cell and myeloid cell immunity were evaluated in three cohorts of healthy donors and oncologic patients, including those infected with SARS-CoV-2, BNT162b2-vaccinated (mRNA vaccine), and with previous COVID-19 and subsequently vaccinated. We concluded that vaccination was a poor inductor of cellular immunity towards the S protein. Memory T-cells were only detected in patients and healthy donors with COVID-19 previous to vaccination but with an accentuated Th17 inflammatory profile, together with elevated numbers of circulating neutrophils.

**Abstract:**

It is unclear whether patients with cancer present inherently impaired responses to COVID-19 and vaccination due to their treatments, neoplastic diseases or both. To address this question, immune profiling was performed in three cohorts of healthy donors and oncologic patients: infected with SARS-CoV-2, BNT162b2-vaccinated, and with previous COVID-19 disease and subsequently vaccinated. Cancer patients showed good antibody responses to vaccination, but poor induction of T-cell responses towards the S protein when compared to infection. Following natural infection, the major targets for T-cells were the SARS-CoV-2 structural proteins M and S, but not the N protein. Similar to antibody titers, the T-cell responses quickly decayed after six months post-vaccination. Significant memory T-cell expansion was observed in vaccinated donors only if previously diagnosed with COVID-19 before undergoing vaccination. Oncologic patients with previous COVID-19 followed by vaccination exhibited potent IL-17+ CD4 and CD8 T-cell responses and elevated numbers of circulating neutrophils in peripheral blood.

## 1. Introduction

In December 2019, an outbreak of SARS in Wuhan, China, by severe acute respiratory syndrome coronavirus-2 (SARS-CoV-2) caused the COVID-19 pandemic that is still ongoing [1,2]. Cancer patients are considered a high-risk population for contracting severe COVID-19 disease that could lead to death [3]. Oncologic patients often present co-morbidities and risk factors associated with COVID-19 severity, including older age, chronic inflammation and genetic alterations associated with severe disease [2,3,4,5]. Patients with cancer are usually immunocompromised by the disease and antineoplastic treatments [3,6,7,8]. Another frequent feature in oncologic patients is T-cell senescence. T-cells proceed towards terminal differentiation during aging by a sequential loss of CD27 and CD28 co-receptor surface expression [9,10]. T-cell senescence is characterized by a severe loss of effector functions and impaired anti-viral immunity. Senescent CD27^neg^ CD28^neg^ T-cells are enriched in effector phenotypes such as effector-memory (CD62L^neg^ CD45RA^neg^) and effector T-cells (CD62L^neg^ CD45RA^+^), with a loss of central memory (CD62L^+^ CD45RA^neg^) and naïve (CD62L^+^ CD45RA^+^) phenotypes [10]. In addition, cancers exacerbate chronic inflammation, favouring inflammatory cytokine release that contributes to COVID-19 clinical syndrome [11,12]. It is yet unclear how these alterations impact immunity against SARS-CoV-2 and responses to vaccination [3,13,14].

Immune responses to SARS-CoV-2 infection in healthy subjects are diverse and complex [15,16], and only a few studies have addressed this in oncologic patients. Overall, patients with cancer have shown comparable antibody responses to healthy subjects upon vaccination [13,17,18,19]. However, T-cell responses are usually strongly reduced in patients with cancer [17]. Although regarded as a high-risk population, patients with cancer were underrepresented in clinical trials assessing vaccine safety and efficacy [20,21]. Overall, high seroconversion rates were shown with comparable or slightly lower antibody titres in patients with solid tumours compared to healthy donors [19,20,22,23,24,25,26,27,28,29,30,31,32,33,34]. However, a meta-analysis of 35 studies suggested lower protection by vaccination in oncologic patients [35]. T-cell activities towards the S protein [22,23,29,33] showed variable results which ranged from diminished responses [22] to lower activation rates [23,33]. However, some studies have shown responses comparable to healthy donors [29].

So far, studies addressing SARS-CoV-2 immunity and responses to vaccination in patients with cancer remain scarce [14,35]. For instance, three of the structural proteins (S, M and N) are the main components of the coronavirion [36,37], but only the S protein is included in most vaccine formulations. This could be relevant for vaccine design [38,39]. Finally, it is still far from clear whether the previous infection affects the responses to vaccination in oncologic patients, both in antibody and T-cell responses.

In this study we found that cancer patients with solid tumors present relevant antibody, T-cell and myeloid responses to vaccination. In subjects with previous COVID-19, T-cell responses were boosted following vaccination, with the S and M proteins as the main T-cell targets. Cancer patients also showed baseline inflammation, which could be exacerbated upon infection or following vaccination. Our results indicate that including the S and M proteins as antigens could improve vaccine efficacy by broadening the immune response.

## 2. Materials and Methods

### 2.1. Study Cohort and Design

The study cohort and design are schematically depicted in Figure S1. Peripheral blood samples from 53 healthy donors and 40 cancer patients were obtained in the Oncology Unit of Hospital Universitario de Navarra (HUN) between April and December 2021. Samples corresponded to six study groups, including healthy donors and cancer patients with previous SARS-CoV-2 infection (*n* = 15 and *n* = 10 respectively), vaccinated healthy donors and cancer patients without previous infection (*n* = 18 and *n* = 20 respectively) and vaccinated healthy donors and cancer patients after having an infection (*n* = 10 in both groups). SARS-CoV-2 infection was confirmed by a positive PCR test. A group of unvaccinated healthy donors without previous infection was included as a control (*n* = 10). The total sample size of the study was established a priori to achieve a minimum power of 0.8 considering a large effect size (f = 0.4) using G*Power 3.1 [40]. General clinical characteristics and SARS-CoV-2-related parameters of the study cohort are summarized in Tables S1 and S2, respectively. Infected patients were classified for COVID-19 severity according to the Treatment Guidelines of the NIH (https://www.covid19treatmentguidelines.nih.gov/overview/clinical-spectrum/; accessed on 1 January 2022):

0 = Asymptomatic or Presymptomatic Infection: Positive for SARS-CoV-2 without symptoms.

1 = Mild Illness: Any of the symptoms of COVID-19 without shortness of breath, dyspnoea, or abnormal chest imaging.

2 = Moderate Illness: Evidence of lower respiratory disease during clinical assessment or imaging with oxygen saturation (SpO_2_) ≥94% on room air at sea level.

3 = Severe Illness: Individuals with SpO_2_ <94%, a ratio of arterial partial pressure of oxygen to fraction of inspired oxygen (PaO_2_/FiO_2_) <300 mm Hg, a respiratory rate >30 breaths/min, or lung infiltrates >50%.

4 = Critical Illness: Respiratory failure, septic shock, and/or multiple organ dysfunction.

### 2.2. Sample Processing, PBMCs Restimulation and Flow Cytometry

Blood collection, peripheral blood mononuclear cells (PBMCs), myeloid cells and T-cell purification, activation and flow cytometry were carried out as previously described [41]. The following fluorochrome-conjugated antibodies were used: CD14-Violet Fluor 450 (Ref 75-0149-T100, TONBO, San Diego, CA, USA), CD11b-PerCP-Cy5-5 (Ref 65-0112-U1, TONBO), CD62L-APC (Ref 130-113-617, Miltenyi, North Rhine-Westphalia, Germany), CD66b-APC-Cy7 (Ref 130-120-146, Miltenyi), CD54-FITC (Ref 130-104-214, Miltenyi), CD19-PE (Ref 130-113-731, Miltenyi), CD3-APC (Ref 130-113-135, Miltenyi), CD8-APC-Cy7 (Ref 130-110-681, Miltenyi), CD4-FITC (Ref 130-114-531, Miltenyi), CD27-PE (Ref 50-0279-T100, TONBO), CD28-PE-Cy7 (Ref 130-126-316, Miltenyi). CD8-PE-Cy7 (Ref 130-110-680, Miltenyi), CD4-APC-Cy7 (Ref 25-0049-T100, TONBO), CD154-PerCP-Cy5-5 (Ref 130-122-800, Miltenyi), CD137-PE (Ref 130-110-763, Miltenyi), IFNγ-FITC (Ref 130-113-497, Miltenyi), IL-17-Vio770 (Ref 130-118-249, Miltenyi), CD45RA-FITC (Ref 35-0458-T025, TONBO), CD62L-APC (Ref 130-113-617, Miltenyi).

For T-cell activation, half a million PBMC cells per well were plated in a 96-well plate, and restimulated with 0.8 ng/µL of the following SARS-CoV-2 PepTivators (Miltenyi) separately: PepTivator SARS-CoV-2 Prot_M, PepTivator SARS-CoV-2 Prot_N, PepTivator SARS-CoV-2 Prot_S, PepTivator SARS-CoV-2 Prot_S1, PepTivator SARS-CoV-2 Prot_S+. S protein PepTivators were mixed for restimulations. Cells were incubated for 17–19 h at 37 °C and then treated with 1 µL/mL of Brefeldin A for 4 h (ThermoFisher Scientific, Waltham, MA, USA). Cells were washed and stained for flow cytometry. Control paired DMSO-mock treated PBMCs were carried out to remove non-specific background from T-cell stimulations.

### 2.3. SARS-CoV-2 Protein Expression and Purification

For enzyme-linked immunoassay (ELISA) and restimulation studies M, S and N proteins were produced using Bac-to-Bac baculovirus expression. Briefly, synthetic genes encoding S1 (1–303 amino acid), full-length N and the cytoplasmic domain of the M protein (1–100 amino acid) were fused to histidine tags and cloned. Protein production and purification by Ni-NTA affinity and size exclusion chromatographies were performed following standard protocols (Bac-to-Bac, Thermofisher).

### 2.4. Enzime Linked Immunosassay (ELISA)

Donor sera were obtained from peripheral blood, centrifuged and frozen at −20 °C. For detection of S- and N-specific antibodies, a 96-well plate was coated with 5 µg/mL of the corresponding SARS-CoV-2 protein, followed by blocking with PBS-2% bovine serum albumin (BSA, Ref A9647-100G, Merck, Frankfort, Germany.). Three sera dilutions (1:800, 1:250 and 1:80) were used to detect anti-S antibodies, anti-N antibodies and anti-M antibodies, respectively. Anti-human IgG HRP-labelled antibody (ThermoFisher) was used as a secondary antibody. ELISAs were developed with 100 µL TMB substrate (Sigma, St. Louis, MO, USA) and read at 450 nm.

### 2.5. Statistical Analyses

Statistical analyses were performed with GraphPad 8. Variables under study were tested for normality (Kruskal–Wallis test), homogeneity of variances (F test), and homogeneity (Spearman’s coefficient of variation). Antibody titres and percentages of cell types as quantified by flow cytometry were either not normally distributed or showed high variability. Hence, for multi-group comparisons of these variables, non-parametric Kruskal–Wallis tests were performed, followed by pair-wise comparisons with Dunn’s test. For experiments involving only two independent groups, the non-parametric Mann–Whitney U-test was used. The percentages of T-cell phenotypes were normally distributed, homogeneous and with comparable variances. In this case, one-way ANOVAs were carried out, followed by a posteriori pair-wise comparisons with Tukey’s test.

### 2.6. Study Approval

This study was conducted according to the principles of the Declaration of Helsinki. The study and informed consent documents were approved by the Clinical Research Ethics Committees of Hospital Universitario de Navarra (Comité ético de investigación clínica, protocol number approval PI_2020/47). Informed consent was obtained from the subjects.

## 3. Results

### 3.1. Cohort Characteristics and Study Design

An exploratory study was performed on a small cohort of cancer patients undergoing current clinical anti-cancer treatments who received SARS-CoV-2 vaccines. The overall aim was to test the efficacy of current mRNA vaccines to raise antibody and T-cell responses in cancer patients compared to healthy donors and characterize the immunological profile of these responses. The total sample size of the study was established to achieve a minimum power of 0.8 considering a large effect size (f = 0.4) using G*Power 3.1 [40]. Clinical and SARS-CoV-2-related characteristics of the cohorts are summarized in Tables S1 and S2. Most cancer patients with solid tumours were under anti-neoplastic treatments, i.e., mostly chemotherapy, at the time of sample collection. Treatments were not interrupted during vaccination. The degree of COVID-19 severity was generally higher in cancer patients compared to healthy donors (Figure S2A,B). The majority of donors were vaccinated with the mRNA vaccine BNT162b2 expressing the Wuhan strain S protein (Pfizer). The time elapsed from the time of SARS-CoV-2 infection or vaccination to sample collection was heterogeneous, as all of samples were retrieved during treatments (Figure S2D,E). However, these times were significantly different between the unvaccinated cancer patients and vaccinated cancer patients with a previous occurrence of COVID-19. For the latter group, vaccination had to be delayed until the full resolution of the disease, following the current clinical guidelines. 45.5% of healthy vaccinated donors completed the vaccination regime more than six months before sample extraction.

This study found comparable responses to vaccination independently of the type of solid tumor and no significant interactions were found between vaccination and sex.

### 3.2. Profiling of Antibody Responses towards S, M and N Proteins

**Figure 1:**
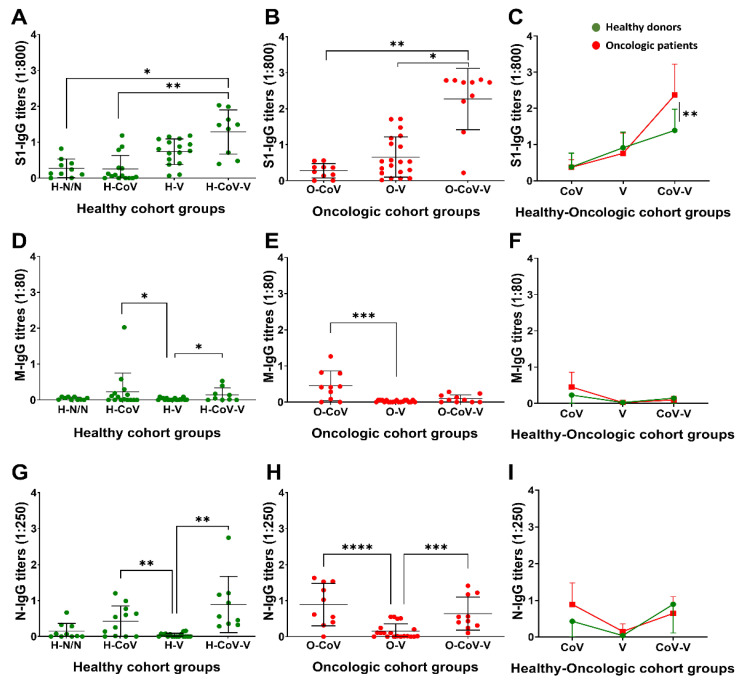
IgG antibody titres against S, M and N proteins. Backgrounds from paired technical controls were removed from each sample. (**A**–**C**) S-specific IgG antibody titres in sera (1:800) from healthy donors and oncologic patients. (**D**–**F**) M-specific IgG antibody titres in sera (1:80) from healthy donors and oncologic patients. (**G**–**I**) N-specific IgG antibody titres in sera (1:250) from healthy donors and oncologic patients. (**A**,**B**,**D**,**E**,**G**,**H**) Non-parametric Kruskal–Wallis test was used for multiple comparisons followed by Dunn’s test for selected pair-wise comparisons. (**C**,**F**,**I**) Pair-wise comparisons were performed using the Mann–Whitney U-test. H-N/N, non-vaccinated, non-COVID-19 donors; H-CoV, healthy donors with previous COVID-19 infection; H-V, vaccinated healthy donor; H-CoV-V, vaccinated healthy donor with previous COVID-19; O-CoV, oncologic patient with previous COVID-19; O-V, vaccinated oncologic patients; O-CoV-V, vaccinated oncologic patients with previous COVID-19; *, **, *** and **** indicate significant (*p* < 0.05), very significant (*p* < 0.01), highly significant (*p* < 0.001) and very highly significant (*p* < 0.0001) differences, respectively.

IgG antibody titers were evaluated towards the S, M and N proteins, considering that M and N responses would be markers of previous COVID-19 disease. In general terms, donors previously diagnosed with COVID-19 presented low S-specific IgG titres unless vaccinated (Figure 1A,B). Vaccination in these donors highly elevated the S-specific IgG titers compared to the donors vaccinated without previous COVID-19, indicating an enhancing effect on the vaccination (Figure 1A,B). Within these groups, S-specific titres were elevated in cancer patients following vaccination compared to the corresponding group of healthy donors (Figure 1C). As a positive correlation between the antibody titres and disease severity was described before [16], we tested if this was the case in our cohorts. COVID-19 severity did not reach statistical significance in our study between these groups (Figure S2C). Otherwise, although the time elapsed from vaccination to sample collection was shorter in patients with cancer, this difference did not reach statistical significance either (Figure S2E). Then, the IgG titres were quantified in vaccinated healthy donors as a function of the time elapsed from vaccination to sample collection. There was a tendency for reduced IgG titres in donors who completed their vaccination regimen more than six months before sample collection, but this did not reach statistical significance either (Figure S3).

We expected to find antibody responses toward M and N proteins only in donors with previous SARS-CoV-2 infection. M-specific IgG antibody titres were mildly elevated in donors with previous infection (Figure 1D,E), without differences between healthy donors and oncologic patients (Figure 1F). In contrast, N-specific IgG titres were significantly elevated (Figure 1G,H), without differences between healthy and cancer patients (Figure 1I).

### 3.3. Profiling of CD4 T-Cell Activation and Differentiation Phenotypes

**Figure 2:**
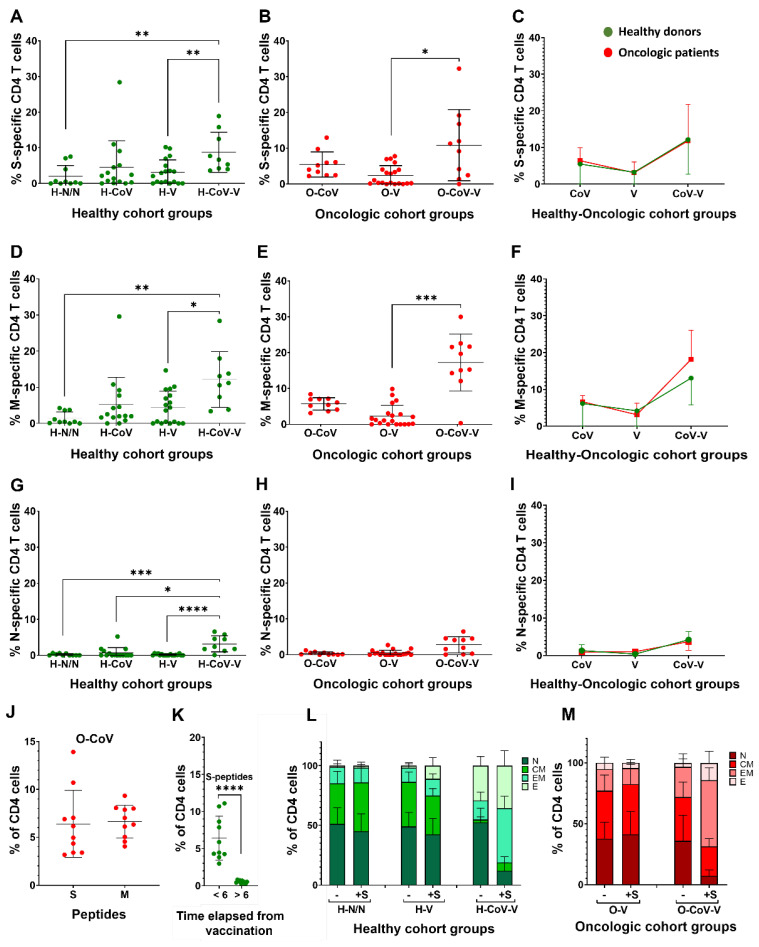
CD4 T-cell responses to S, M and N peptides of SARS-CoV-2 proteins. Paired backgrounds from technical controls were removed from the data. (**A**–**C**) Percentage CD4 T-cells in PBMCs stimulated with S peptides in healthy donors and oncologic patients. (**D**–**F**) Percentage CD4 T-cells in PBMCs stimulated with M peptides in healthy donors and oncologic patients. (**G**–**I**). Percentage CD4 T-cells in PBMCs stimulated with N peptides in healthy donors and oncologic patients. (**A**,**B**,**D**,**E**,**G**,**H**) Statistical significance was tested by Kruskal–Wallis tests, followed by Dunn’s pair-wise comparison tests. (**J**) Dot plot of the percentage of S and M-specific CD4 T-cells. (**K**) Dot plot of S-specific CD4 T-cells from samples collected at the indicated timelines after completion of vaccination regimes. (**C**,**F**,**I**,**J**,**K**) Pair-wise comparisons were performed using the Mann–Whitney U-test. (**L**,**M**) Relative percentages of CD4 T-cell differentiation phenotypes in healthy donors and oncologic patients. Detailed statistical differences are shown in Figure S5. N, CM, EM and E, indicate naïve-stem cell (CD62L+ CD45RA+), central memory (CD62L^+^ CD45RA^neg^), effector memory (CD62L^neg^ CD45RA^neg^) and effector (CD62L^neg^ CD45RA^+^) phenotypes. H-N/N—non-vaccinated, non-COVID-19 donors; H-CoV—healthy donors with previous COVID-19 infection; H-V—vaccinated healthy donor; H-CoV-V—vaccinated healthy donor with previous COVID-19; O-CoV—oncologic patient with previous COVID-19; O-V—vaccinated oncologic patients; O-CoV-V—vaccinated oncologic patients with previous COVID-19; *, **, *** and **** indicate significant (*p* < 0.05), very significant (*p* < 0.01), highly significant (*p* < 0.001) and very highly significant (*p* < 0.0001) differences, respectively.

Systemic T-cell responses toward the three main structural proteins were then evaluated. PBMCs were incubated with viral protein-specific peptivators and upregulation of the activation markers CD154 and CD137 assessed by flow cytometry (Figure S4). Peptivators consist of pools of 15-mer peptides with 11 amino acids that overlap and cover the SARS-CoV-2 proteins. This ensures the evaluation of T-cells specific for the three main proteins of SARS-CoV-2. S-specific CD4 T-cells were detectable in patients with previous SARS-CoV-2 infection, especially in cancer patients. However, vaccination alone was not a potent inductor of S-specific CD4 T-cells. In contrast, the CD4 T-cell responses towards the S protein were boosted in vaccinated groups with previous COVID-19, suggesting an immune enhancer effect on the T-cell responses (Figure 2A,B). No differences were observed between healthy donors and cancer patients (Figure 2C).

We expected to find M- and N-specific T-cell responses in donors with previous SARS-CoV-2 infection. M-specific CD4 T-cells were most abundant in vaccinated donors with previous COVID-19 without differences between healthy and cancer patients (Figure 2D–F). Unexpectedly, some CD4 reactivity towards M protein was observed in vaccinated healthy donors and oncologic patients, who did not reportedly have a previous infection. The N protein was a poor inductor of T-cell responses in our cohorts, and these were only detected in vaccinated donors with previous infection (Figure 2G–I).

Overall, we observed that in donors with previous COVID-19, both S and M proteins were equally good targets for CD4 responses (Figure 2J). However, in vaccinated donors, these responses decayed six months post-vaccination (Figure 2K).

Most cancer patients present dysfunctional T-cell immunity characterised by altered T-cell phenotypes [42]. Therefore, we investigated the differentiation of CD4 T-cell phenotypes in our cohorts by assessing CD62L and CD45RA expression profiles (Figure S5A). Vaccination caused a significant increase in effector cells (CD62L^neg^ CD45RA^+^) in healthy donors (Figure 2L and Figure S5B). Importantly, donors with previous COVID-19 showed a significant increase in effector memory (CD62L^neg^ CD45RA^neg^) and effector (CD62L^neg^ CD45RA+) S-specific CD4 T-cells following vaccination. Our results also showed that having a previous SARS-CoV-2 infection followed by vaccination expanded memory CD4 T-cells (Figure 2L,M and Figure S5C–F). No significant differences were observed in CD27/CD28 expression profiles in T-cells between healthy and cancer donors (Figure S5G,H).

### 3.4. Profiling of CD8 T-Cell Activation and Differentiation Phenotypes

**Figure 3:**
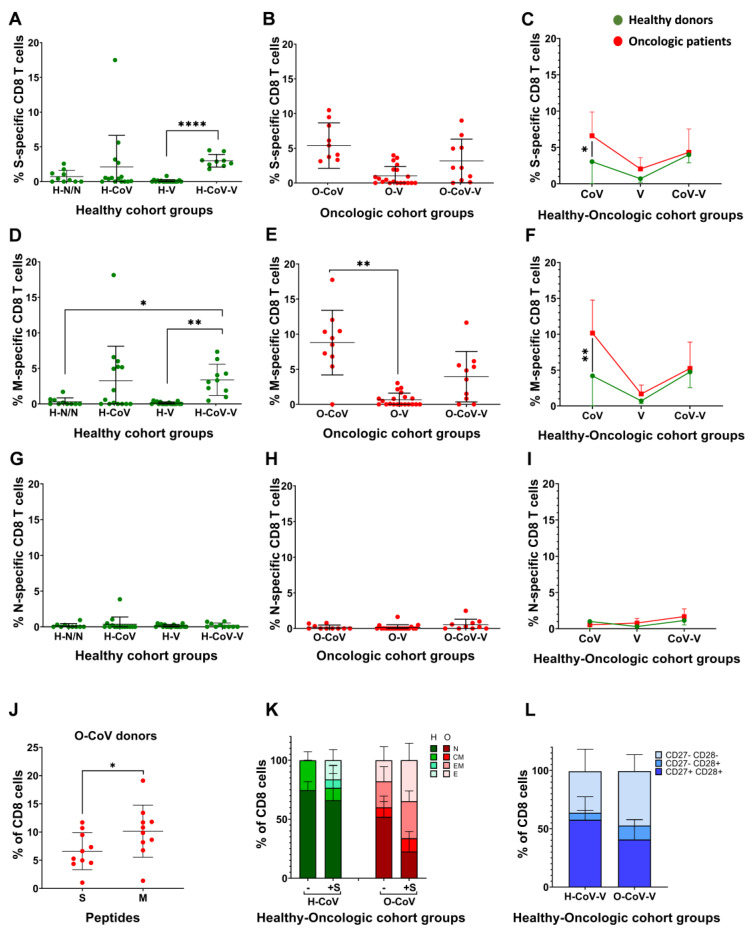
CD8 T-cell responses to S, M and N peptides of SARS-CoV-2 proteins. Paired backgrounds from technical controls were removed from the data. (**A**–**C**) Percentage of S-specific CD8 T-cells in PBMCs stimulated with S-peptides in healthy donors and oncologic patients. (**D**–**F**) Percentage of S-specific CD8 T-cells in PBMCs stimulated with M-peptides in healthy donors and oncologic patients. (**G**–**I**) Percentage of S-specific CD8 T-cells in PBMCs stimulated with N-peptides in healthy donors and oncologic patients. (**A**,**B**,**D**,**E**,**G**,**H**) Significance was tested with Kruskal–Wallis tests, followed by Dunn’s test. (**J**) Percentage of activated CD8 T-cells after stimulation with S- or M-specific peptides in O-CoV donors. (**C**,**F**,**I**,**J**) U of Mann-Whitney was used to test for significance. (**K**) Relative percentages of CD8 T-cell differentiation phenotypes in the indicated groups of healthy donors and oncologic patients. Means and error bars (standard deviations) are shown. N, CM, EM and E, indicate naïve-stem cell (CD62L+ CD45RA+), central memory (CD62L+ CD45RA^neg^), effector memory (CD62L^neg^ CD45RA^neg^) and effector (CD62L^neg^ CD45RA+) phenotypes. Relevant statistical comparisons are detailed in Figure S8. (**L**) Relative percentages of CD8 T-cell differentiation phenotypes in the indicated groups of healthy donors and oncologic patients according to CD27^neg^ CD28 expression profiles. CD27^+^ CD28^+^, CD27^neg^ CD28+ and CD27^+^ CD28^+^ indicate poorly differentiated, intermediate differentiated and highly differentiated T-cell phenotypes. H-N/N—non-vaccinated, non-COVID-19 donors; H-CoV—healthy donors with previous COVID-19 infection; H-V—vaccinated healthy donor; H-CoV-V—vaccinated healthy donor with previous COVID-19; O-CoV—oncologic patient with previous COVID-19; O-V—vaccinated oncologic patients; O-CoV-V—vaccinated oncologic patients with previous COVID-19; *, ** and **** indicate significant (*p* < 0.05), very significant (*p* < 0.01) and very highly significant (*p* < 0.0001) differences, respectively.

We studied systemic CD8 responses towards the three main structural proteins, by incubating PBMCs with viral protein-specific peptivators and assessing the upregulation of the activation markers CD154 and CD137 within CD8 T-cells by flow cytometry. The strongest CD8 T-cell responses were detected in donors with previous COVID-19 (Figure 3A–C and Figure S6). Unvaccinated cancer patients with previous COVID-19 with higher degree of disease severity (Figure S2B), showed significantly increased S-specific CD8 T-cells compared to their healthy counterpart group (Figure 3C). We also observed significantly elevated percentages of M-specific CD8 T-cells in donors with previous COVID-19 compared to the corresponding group of healthy donors (Figure 3D–F).

No N-specific CD8 T-cells were detected (Figure 3G–I), concluding that during SARS-CoV-2 infection, the M and S proteins are the main CD8 T-cell targets (Figure 3J). In general terms, no significant differences in S-specific CD8 T-cell phenotypes or changes were observed between vaccinated healthy donors and cancer patients (Figure S7A–F). However, in donors with previous COVID-19 there were marked baseline differences after vaccination (Figure 3K and Figure S7B–G). A large proportion of S-specific CD8 T-cells in healthy donors were poorly differentiated phenotypes (CD62L^+^ CD45RA^+^) before PBMC stimulation with S peptides. In contrast, their oncologic counterparts had expanded effector memory and effector T-cell compartments (Figure 3K and Figure S7H). After stimulation with S peptides, and in contrast to healthy donors, cancer patients further expanded T-cells with effector phenotypes with a drastic reduction of naïve T-cells (Figure 3K and Figure S6I). These results strongly indicated that vaccinated cancer patients with previous COVID-19 had exacerbated effector memory and effector CD8 responses. Indeed, baseline T-cell phenotypes in these patients showed a drastic reduction in poorly differentiated (CD27^+^ CD28^+^) phenotypes compared to non-oncologic donors (Figure 3L and Figure S7J). Within donors without previous COVID-19 infection, no significant differences were found between healthy donors and cancer patients after vaccination (Figure S7K).

### 3.5. Evaluation of Inflammatory Cytokine Expression in T-cells

**Figure 4:**
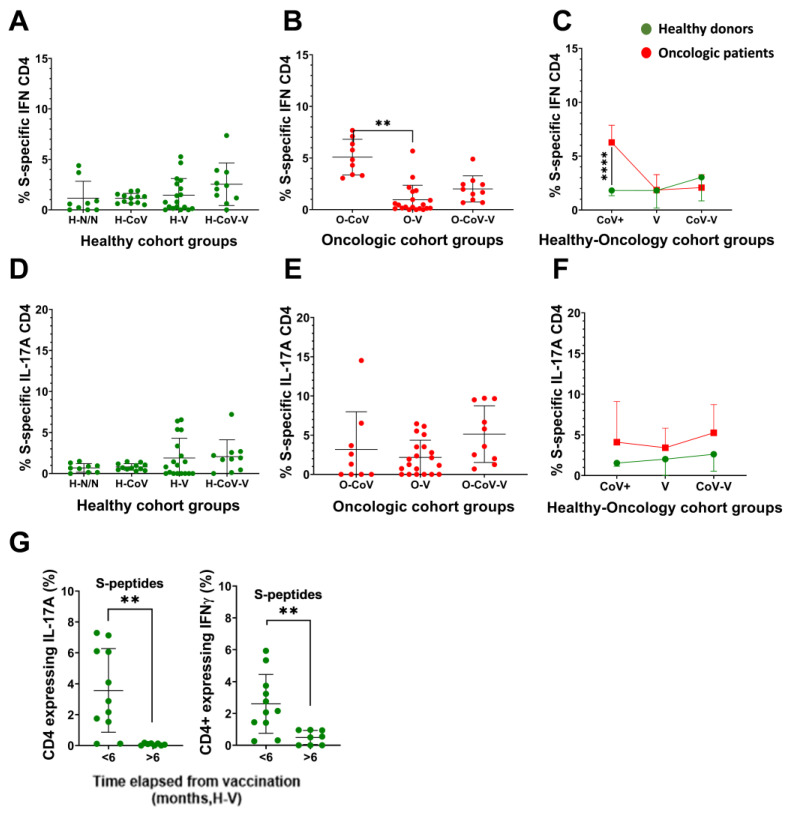
IFNγ and IL-17 expression in S-specific CD4 T-cells. Paired backgrounds from technical controls were removed from the data. (**A**–**C**) Percentage of IFNγ-CD4 T-cells specific for the S protein in healthy donors and oncologic patients. (**D**–**F**) Percentage of IL-17-CD4 T-cells specific for the S protein in healthy donors and oncologic patients. (**A**,**B**,**D**,**E**) Statistical significance was evaluated with the Kruskal–Wallis test, followed by Dunn´s pair-wise comparisons. (**G**) Percentage of CD4 T-cells expressing IL-17 and IFNγ in H-V donors that completed the vaccine regime before sample collection in the indicated timelines. (**C**,**F**,**G**) Significance was tested with the Mann–Whitney U-test. H-N/N—non-vaccinated, non-COVID-19 donors; H-CoV—healthy donors with previous COVID-19 infection; H-V—vaccinated healthy donor; H-CoV-V—vaccinated healthy donor with previous COVID-19; O-CoV—oncologic patient with previous COVID-19; O-V—vaccinated oncologic patients; O-CoV-V—vaccinated oncologic patients with previous COVID-19; ** and **** indicate very significant (*p* < 0.01) and very highly significant (*p* < 0.0001) differences, respectively.

As severe COVID-19 is associated with exacerbated inflammatory responses, IFNγ and IL-17 expression in S-specific CD4 T-cells were evaluated by PBMC stimulation with Peptivators, and evaluation of cytokine expression by intracellular flow cytometry within T-cells (Figure S8). Overall, we found heterogeneity in our cohort, but donors with previous COVID-19 infection had increased proportions of INFγ-CD4 T-cells specific for the S protein especially after vaccination, with the exception of unvaccinated cancer patients (Figure 4A,B). Significantly elevated percentages were found in unvaccinated cancer patients with previous COVID-19 compared to the corresponding healthy group (Figure 4C). IL-17-CD4 T-cells specific for the S protein were elevated in vaccinated donors, suggesting that the mRNA vaccine was an inducer of Th17 responses (Figure 4D,E). Cancer patients showed a trend towards increased IL-17-CD4 T-cells compared to healthy donors, without reaching significance (Figure 4F). Overall, these results indicated an accentuated Th17 response in oncologic donors compared to healthy donors (Figure S2B). In agreement with our previous results, inflammatory S-specific CD4 T-cell subsets decayed six months after vaccination (Figure 4G).

**Figure 5:**
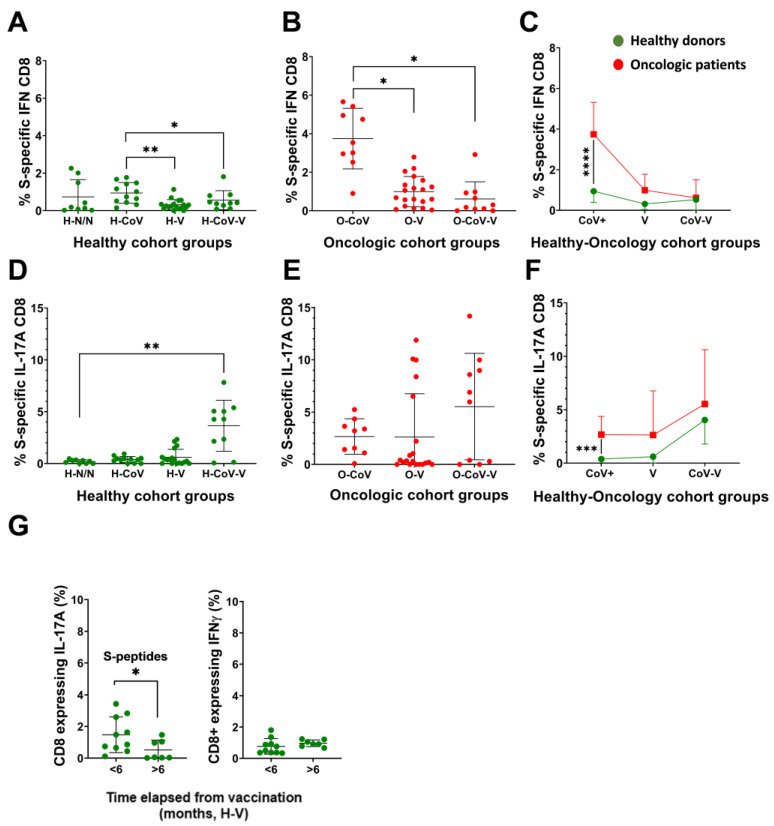
IFNγ and IL-17 expression in CD8 T-cells specific for the S protein**.** Paired backgrounds from technical controls were removed from the data. (**A**–**C**) Percentage of IFNγ-CD8 T-cells specific for the S protein in healthy donors and oncologic patients. (**D**–**F**) Percentage of IL-17-CD8 T-cells specific for the S protein in healthy donors and oncologic patients. (**A**,**B**,**D**,**E**) Statistical significance was evaluated with the Kruskal–Wallis test, followed by Dunn´s pair-wise comparisons. (**G**) Percentage of CD8 T-cells expressing IL-17 and IFNγ expression in H-V donors that completed the vaccine regime before sample collection in the indicated timelines. (**C**,**F**,**G**) Significance was tested with the Mann–Whitney U-test. H-N/N—non-vaccinated, non-COVID-19 donors; H-CoV—healthy donors with previous COVID-19 infection; H-V—vaccinated healthy donor; H-CoV-V—vaccinated healthy donor with previous COVID-19; O-CoV—oncologic patient with previous COVID-19; O-V—vaccinated oncologic patients; O-CoV-V—vaccinated oncologic patients with previous COVID-19; *, **, *** and **** indicate significant (*p* < 0.05), very significant (*p* < 0.01), highly significant (*p* < 0.001) and very highly significant (*p* < 0.0001) differences, respectively.

Inflammatory S-specific CD8 T-cell subsets were quantified by PBMC stimulation with Peptivators and evaluation of cytokine expression by intracellular flow cytometry within T-cells (Figure S8B). Infection but not vaccination was the strongest inducer of S-specific IFNγ-CD8 T-cells in healthy and oncologic patients (Figure 5A,B). We also observed significantly increased percentages of S-specific CD8 T-cells expressing IFNγ in unvaccinated oncologic patients with previous COVID-19 compared to their healthy counterpart group (Figure 5C). There were however marked differences in IL-17-CD8 T-cells, which were increased in subjects with previous COVID-19 following vaccination (Figure 5D,E). In general terms, we observed increased IL-17-CD8 T-cell responses in cancer versus healthy donors, reaching statistical significance again between unvaccinated groups with previous infection (Figure 5F). As with CD4 T-cells, inflammatory S-specific CD8 T-cells decayed six months after vaccination (Figure 5G).

### 3.6. Profiling of Systemic Myeloid Cell Subsets

**Figure 6:**
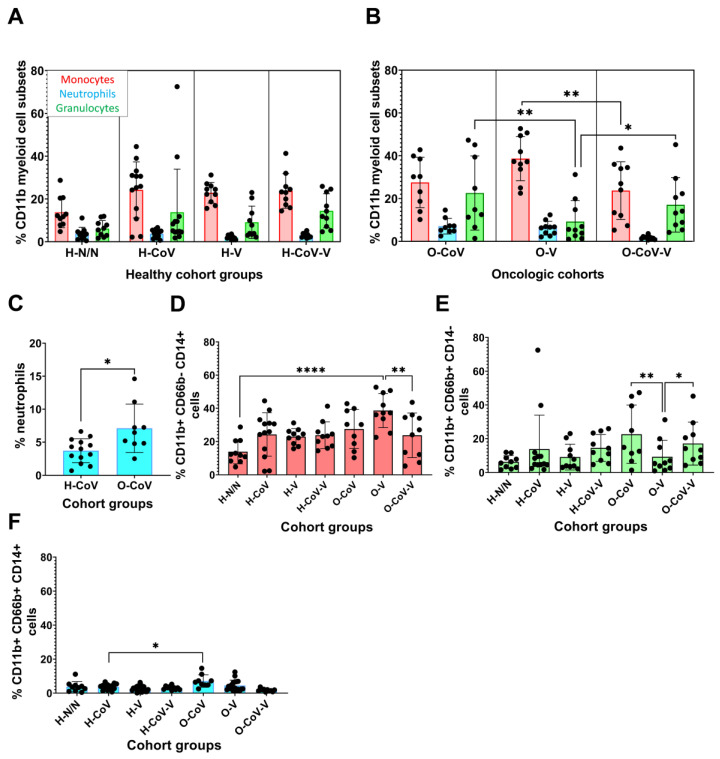
Systemic compositions of myeloid cell subsets. (**A**,**B**) Percentages of monocytes (CD11b^+^ CD66b^neg^ CD14^+^; red), neutrophils (CD11b^+^ CD66b^+^ CD14^+^; blue) and total granulocytes (CD11b^+^ CD66b^+^ CD14^neg^, green), within CD11b^+^ cells. (**C**) Percentage of neutrophils in H-CoV and O-CoV. (**D**–**F**) Percentages of monocytes (CD11b^+^ CD66b^neg^ CD14^+^), neutrophils (CD11b^+^ CD66b^+^ CD14^+^) and granulocytes (CD11b^+^ CD66b^+^ CD14^neg^) in the indicated groups. Statistical significance was evaluated by the Kruskal–Wallis test, followed by Dunn´s pair-wise comparisons. H-N/N—non-vaccinated, non-COVID-19 donors; H-CoV—healthy donors with previous COVID-19 infection; H-V—vaccinated healthy donor; H-CoV-V—vaccinated healthy donor with previous COVID-19; O-CoV—oncologic patient with previous COVID-19; O-V—vaccinated oncologic patients; O-CoV-V—vaccinated oncologic patients with previous COVID-19; *, ** and **** indicate significant (*p* < 0.05), very significant (*p* < 0.01) and very highly significant (*p* < 0.0001) differences, respectively.

We previously showed in lung cancer patients that myeloid profiles enriched in granulocytes contribute to dysfunctional immunity and treatment failure [43,44,45,46]. Therefore, we evaluated whether cancer patients in our cohort showed dysfunctional myeloid profiles that could explain accentuated inflammatory responses to COVID-19 and mRNA vaccination. The percentages of monocytes, granulocytes and neutrophils were quantified in peripheral blood and no differences were found in healthy donors (Figure 6A–C). However, cancer patients with previous COVID-19 showed an elevation of granulocytes compared to vaccinated cancer patients without previous COVID-19 (Figure 5B). Indeed, vaccinated cancer patients without previous COVID-19 showed a significant increase in circulating monocytes. These results suggested that while SARS-CoV-2 infection caused an increase in granulocytes, and particularly neutrophils, vaccination targeted the monocytic lineage in patients without a previous occurrence of COVID-19 (Figure 6B–F).

## 4. Discussion

Cancer patients usually have a compromised immunity from cancer progression and treatments [10,47], which may impact responses to COVID-19 and vaccination. In this work, we carried out an exploratory study with a limited cohort of patients with solid tumors who had been vaccinated, infected with SARS-CoV-2 or both. As such, the sample size for each group was selected to achieve a minimum power of 0.8. As controls, we included several groups of healthy donors. Although limited by the number of subjects in our cohort and the inclusion of cancer patients with distinct solid tumors, we found that our results were comparable, complemented the findings of other studies [28,29,30,31,32,33] and novel conclusions could be derived. Our study broadened the scope to include the assessment of S-, M- and N-specific T-cell responses and myeloid cell signatures, which are relevant in donors with previous COVID-19 infection. Indeed, our results could be important for the design of novel, more efficacious vaccines. Most donors had been vaccinated with the mRNA BNT162b2 vaccine that encodes the Wuhan strain S protein, which is a potent inducer of S-specific antibodies [48,49,50]. However, this vaccine lacks the other two main components of the coronavirion, the M and N proteins. In our study, we included the evaluation of antibody and T-cell responses towards these two latter proteins in donors who had previous COVID-19.

Our results supported studies that found that antibody responses were not impaired in cancer patients [51], but that were limited in time [52]. An important finding in our study was that T-cell responses after vaccination were mainly of effector type and quickly decayed after six months. Memory T-cell expansion was observed in vaccinated patients with a previous occurrence of COVID-19. Hence, vaccination could confer longer protection from reinfections following COVID-19 resolution. This has implications in populations in which infections have been widespread, or following infections and reinfections in vaccinated subjects, as we are observing in the current situation. These observations are especially important for populations at higher risk, such as cancer patients.

Vaccination mainly induced CD4 T-cells, which would be in agreement with the potent antibody responses [53]. In contrast, SARS-CoV-2 infection preferentially expanded CD8 T-cells towards the S and M proteins, in agreement with some studies [54,55]. However, we demonstrate in our study that cancer patients showed even stronger T-cell responses towards the S and M proteins. In our study, we found a mobilization of memory CD4 T-cells responding to SARS-CoV-2 M peptides following vaccination in some donors without previous COVID-19. This result could either reflect that these donors did in fact have asymptomatic COVID-19, or as suggested in other studies, cross-reactivity with T-cells specific for common cold coronaviruses [55,56]. Nevertheless, these results highlight the importance of including M as a vaccination antigen in new vaccine formulations.

The T-cell repertoire of cancer patients was skewed towards differentiated phenotypes expressing IFNγ as shown before [23,57], but even more pronounced towards IL-17 production both in healthy donors and cancer patients. Vaccination induced IFNγ and elevated IL-17 in CD4 T-cells, a marker of Th17 responses [23,57]. Indeed, SARS-CoV-2 infection induced a Th17 signature, which very likely contributes to disease severity through exacerbated inflammation. Apart from this, the mRNA vaccine itself may be promoting Th17 responses, through, for example, inflammasome activation due either to the mRNA content or the liposome carrier. Although we do not provide direct evidence for these hypotheses in our current study, it could be interesting to develop novel mRNA vaccines that contain adjuvants designed for skewing Th17 responses towards the less inflammatory Th1 responses. Indeed, as neutralizing antibodies are key to controlling SARS-CoV-2 infection, vaccine adjuvants that enhance Th2 responses could be of interest. If this can be achieved in current mRNA vaccines, it would very likely improve their efficacy at least for the treatment of strongly inflammatory infectious diseases such as COVID-19. Otherwise, the use of alternative protein-based vaccines could be favored, as they are more amenable for the incorporation of different injectable vaccine adjuvants with variable immunomodulatory properties.

Finally, we confirmed that the cancer patients in our cohort showed elevated percentages of circulating neutrophils, which is a signature of dysfunctionality and elevated baseline inflammation as previously shown by us [43,46,47]. This could explain the exacerbated Th17 responses after vaccination and/or SARS-CoV-2 infection in cancer patients. Elevated neutrophil counts are frequent in oncologic patients, but we found this to be accentuated even in the subjects with previous COVID-19.

## 5. Conclusions

Cancer patients with solid tumors exhibit proficient antibody, T-cell and myeloid responses to vaccination. In subjects with previous COVID-19, T-cell responses are boosted after vaccination, with the S and M proteins as the main T-cell targets. These results strongly suggest that including M protein as a vaccine antigen could improve vaccine efficacy. Cancer patients also showed evidence of baseline inflammation, which can be exacerbated upon infection or following vaccination that elicits Th17 responses. This is an important issue that requires further research in novel mRNA vaccine adjuvants that can skew a Th17 response towards Th1 and Th2 responses.

Nevertheless, our study presents some limitations due to logistical reasons. We had to group together patients with different types of solid tumors. This, in addition, introduces the different anti-neoplastic treatments as an uncontrolled variable. Although the immunological results were rather homogeneous compared to healthy donors, more accurate results could be derived by carrying out a follow-up study on specific solid tumors and homogeneous treatments.

Therefore, further studies on the consequences of vaccination and infection in cancer patients are merited. Our results also indicate that including the S and M proteins as antigens could improve vaccine efficacy.

## Data Availability

The data presented in this study are available in this article (and supplementary material).

## Supplementary Materials

The following supporting information can be downloaded at: https://www.mdpi.com/article/10.3390/cancers14184464/s1, Table S1: Clinical and demographic characteristics of the study cohort; Table S2: SARS-CoV-2- related parameters of the study cohort; Figure S1: Schematic representation of the study cohort and design; Figure S2: COVID-19 severity degree and time elapsed from SARS CoV-2 infection and/or vaccination to sample collection; Figure S3: Dynamics of S-specific IgG titres; Figure S4: Flow cytometry and gating strategy for quantification of activated CD4 T-cells; Figure S5: CD4 T-cell phenotypes after stimulation with S peptides; Figure S6: Flow cytometry and gating strategy for quantification of activated CD8 T-cells; Figure S7: Differentiation phenotypes in CD8 T-cells; Figure S8: Flow cytometry and gating strategy for quantification of cytokine expression within activated T-cells.
