# Supplementary material for: Immune Profiling Uncovers Memory T-Cell Responses with a Th17 Signature in Cancer Patients with Previous SARS-CoV-2 Infection Followed by mRNA Vaccination
Source: Cancers (Basel). 2022 Sep 14;14(18):4464. doi: 10.3390/cancers14184464 (PMC9496802; doi:10.3390/cancers14184464)
Supplement: Supplementary file 1 [file cancers-14-04464-s001.zip › cancers-1896077-supplementary.pdf]

Table S1. Clinical and demographic characteristics of the study cohort

		Healthy donors				Oncologic patients		
		H-N/N (n= 10)	H-CoV (n= 15)	H-V (n = 22)	H-CoV-V (n= 10)	O-CoV (n = 10)	O-V (n= 20)	O-CoV-V (n =10)
Age (median, Q1,Q3)		40 (25-58)	55 (53-61)	55 (42-61)	48 (34-61)	68 (61-75)	65 (60-71)	68 (57-74)
Gender (n, %)	Male	6 (60)	9 (60)	2 (9)	2 (20)	8 (80)	8 (40)	5 (50)
	Female	4 (40)	6 (40)	20 (91)	8 (80)	2 (20)	12 (60)	5 (50)
Comorbidities (n, %)	AHT	1 (11)	4 (29)	2 (9)	1 (10)	4 (40)	8 (40)	6 (60)
	DM	0 (0)	0 (0)	0 (0)	1 (10)	3 (30)	3 (15)	3 (30)
	DLP	1 (11)	5 (36)	4 (18)	2 (20)	7 (70)	7 (35)	3 (30)
	IC	0 (0)	0 (0)	0 (0)	0 (0)	3 (30)	3 (15)	0 (0)
	Neumo	0 (0)	3 (21)	1 (4,5)	0 (0)	5 (50)	4 (20)	1 (10)
Type of cancer (n, %)						Colon/ rectum 4 (40); breast 2 (20); pancreas, meso., lung, sarcoma 1 (10)	Pancreas 8(40); liver 5 (25); colon/rectum 3 (15); ovary 2 (10); renal, gastric 1 (5)	Breast, pancreas, gastric 2 (20); meso., lung, ovary, salivary glands 1 (10)
Cancer treatment during sample extraction (n, %)	NT					2 (20)	5 (25)	0 (0)
	CT					5 (50)	10 (50)	5 (50)
	IT					0 (0)	0 (0)	1 (10)
	TKI					2 (20)	2 (10)	2 (20)
	CT+TKI					1 (20)	3 (15)	2 (20)

CoV, SARS CoV-2 infection; vac, vaccinated; CT, chemotherapy; DLP, dyslipidaemia; DM, diabetes mellitus; AHT, arterial hypertension; IC, ischemic cardiopathy; IT, immunotherapy; meso., mesothelioma; Neumo., neumopathies; NT, non-treated; TKI, tyrosin-kinase inhibitors; Q1, quartile 1; Q3, quartile 3; vac, vaccinated.

Table S2. SARS CoV-2- related parameters of the study cohort

		Healthy donors				Oncologic patients		
		H-N/N (n= 10)	H-CoV (n= 15)	H-V (n = 22)	H-CoV-V (n= 10)	O-CoV (n = 10)	O-V (n= 20)	O-CoV-V (n =10)
SARS CoV-2 infection severity	0		4(27)		5 (56)	0 (0)		3 (30)
	1		11(73)		4(44)	3(30)		5(50)
	2		0(0)		0(0)	3(30)		0(0)
	3		0(0)		0(0)	4(40)		2(20)
Clinical manifestation	Pneum.		0(0)		0(0)	9(90)		3(30)
	Complications		0 (0)		0(0)	4 (40)		0(0)
Time: infection-sample months (mean±SD)			4.7±2.4		8.5±4.2	2.8±2.6		8.6±3.3
Cancer treatment during SARS CoV-2 infecction (n, %)	NT					3 (30)		6 (60)
	CT					4 (40)		2 (20)
	IT					0 (0)		1 (10)
	TKI					2 (20)		1 (10)
	CT+TKI					1 (10)		0 (0)
Type of vaccine (n, %)	BNT162b2 (Pfizer)			18 (82)	9 (90)		15 (75)	10 (100)
	mRNA-1273 (Moderna)			2 (9)	1 (10)			
	Vaxzevria (Astrazeneca)			2 (9)	0 (0)		5 (25)	0 (0)
Adverse events (n, %)				0(0)	0(0)		2 (10)	0 (0)
Time: vaccine-sample months (mean±SD)				5.1±3.7	3.1±1.3		3.3±2.7	0.9±0.4
Cancer treatment during vaccination (n, %)	NT						2 (10)	0 (0)
	CT						11 (55)	2 (30)
	IT						0 (0)	1 (10)
	TKI						3 (11)	3 (30)
	CT+TKI						4 (20)	3 (30)

CoV, SARS CoV-2 infection; vac, vaccinated; CT, chemotherapy; IT, immunotherapy; NT, non-treated; SD, standard deviation; TKI, tyrosin-kinase inhibitors; Q1, quartile 1; Q3, quartile 3; vac, vaccinated.

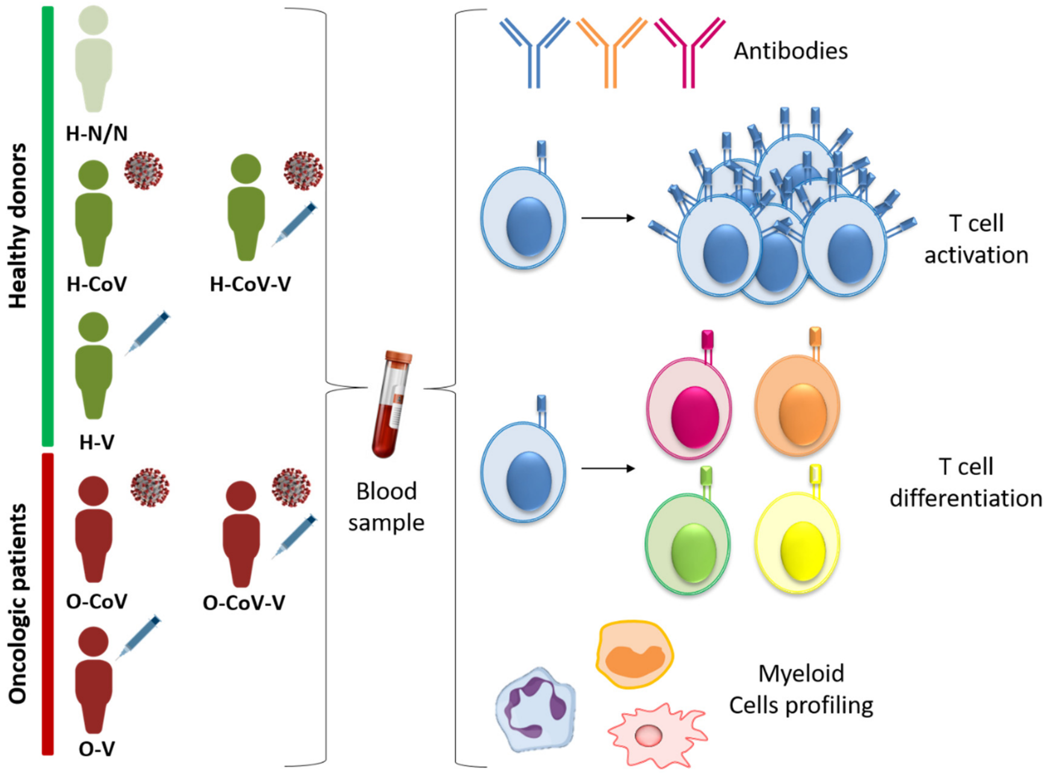

Figure S1. Schematic representation of the study cohort and design. Samples from healthy donors and cancer patients naturally infected by SARS CoV-2 and vaccinated with/without previous SARS CoV-2 infection were collected. A group of non-infected non-vaccinated healthy donors was included as control responses. Blood samples were retrieved following informed consent and analysed for antibody titers, T cell activation and differentiation, and characterization of systemically circulating myeloid cell subsets.

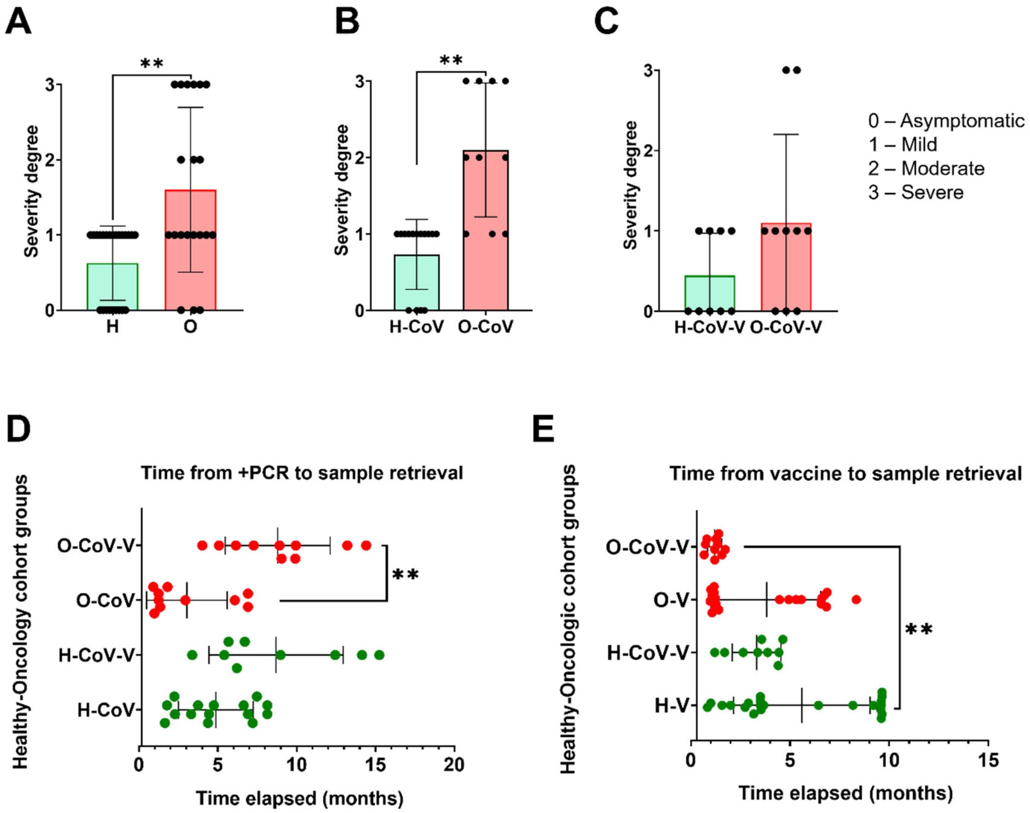

Figure S2. COVID-19 severity degree and time elapsed from SARS CoV-2 infection and/or vaccination to sample collection. **(A, B, C)** COVID-19 severity degree according to the NIH guidelines in all the indicated groups of healthy donors and oncologic patients**.** The U of Mann-Whitney test was used to evaluate significance. **(D, E)** Time elapsed from SARS CoV-2 diagnosis to sample collection and from vaccination to sample collection in the indicated groups of healthy donors and cancer patients. Kruskal-Wallis test was used for multiple comparisons followed by Dunn´s test for pair-wise comparison. H-CoV, healthy donors with previous COVID-19 infection; H-V, vaccinated healthy donor; H-CoV-V, vaccinated healthy donor with previous COVID-19; O-CoV, oncologic patient with previous COVID-19; O-V, vaccinated oncologic patients; O-CoV-V, vaccinated oncolgic patients with previous COVID-19; *, **, *** and **** indicate significant ($p<0.05$) , very significant ($p<0.01$) and highly significant ($p<0.001$) differences, respectively.

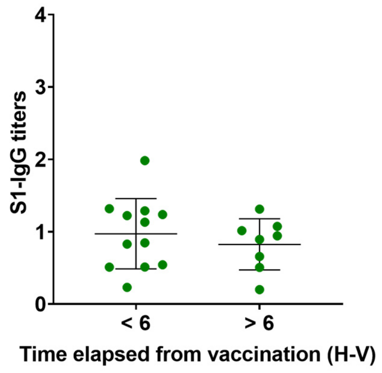

Figure S3. Dynamics of S-specific IgG titres. S specific IgG antibody titres in vaccinated healthy donors without previous infection (V-H) from samples collected less than 6 months and more than 6 months after vaccination. The U of Mann-Whitney test was used for statistical significance giving a non-significant result (P>0.05).

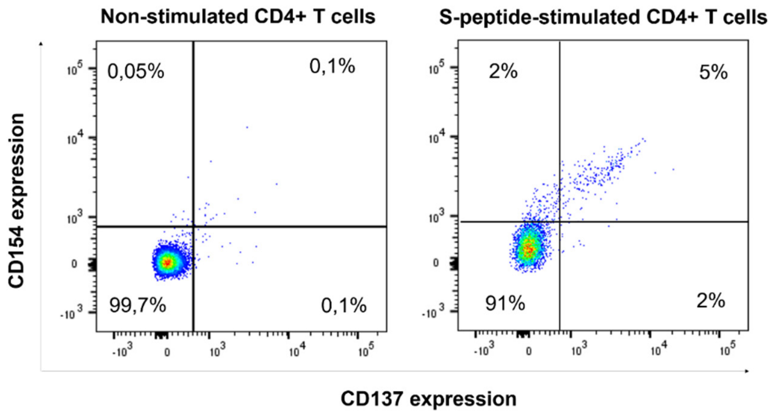

Figure S4. Flow cytometry and gating strategy for quantification of activated CD4 T cells. Representative flow cytometry density plots of CD154 and CD137 co-expression profiles in CD4 T cells from donors before and after stimulation with S-peptides, as indicated.

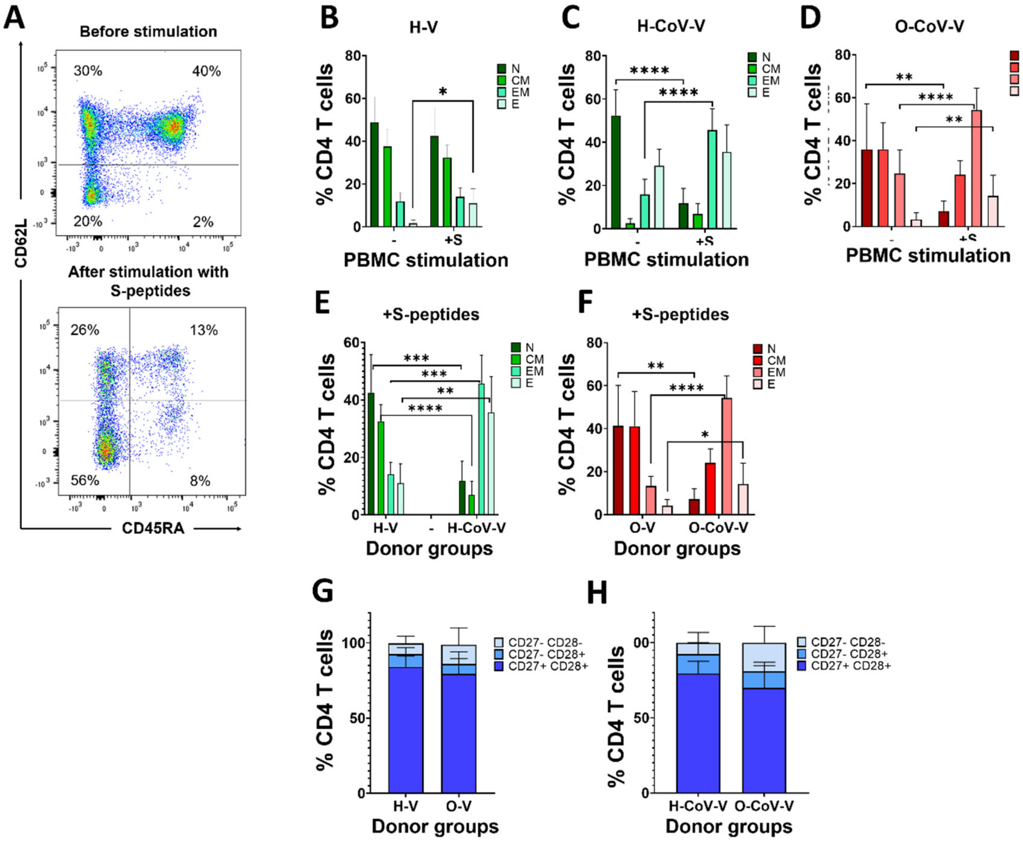

Figure S5. CD4 T cell phenotypes after stimulation with S peptides. **(A)** Representative flow cytometry density plots with CD62L-CD45RA co-expression profiles in CD4 T cells before and after the stimulation with S-peptides. Quadrants were established with unstained controls. Percentages of the corresponding populations are shown within the quadrants. **(B, C)** CD4 T cell phenotypic changes in H-V and H-CoV-V donors. (-) and (+S), non-stimulated and S-peptide stimulation. N, CM, EM and E, indicate naïve-stem cell (CD62L+ CD45RA+), central memory (CD62L+ CD45RA-), effector memory (CD62L- CD45RA-) and effector (CD62L- CD45RA+) phenotypes. **(D)** Phenotypic changes in CD4 T cells within O-CoV-V before and after stimulation with S-peptides. **(E,F)** Effects of previous CoV infection in vaccinated healthy donors and oncologic patients over T cell phenotypes after stimulation with S- peptides. **(B-F)** Relevant statistical comparisons are indicated by ANOVA followed by pair-wise comparisons with Tukey's test. **(G, H)** Relative percentages of CD4 T cell differentiation phenotypes in H-V and O-V and in H-CoV-V and O-CoV-V CD27+ CD28+, CD27- CD28+ and CD27+ CD28+ indicate poorly differentiated, intermediate differentiated and highly differentiated T cell phenotypes. U of Mann-Whitney was used to test for significance. H-N/N, non-vaccinated, non-COVID-19 donors; H-CoV, healthy donors with previous COVID-19 infection; H-V, vaccinated healthy donor; H-CoV-V, vaccinated healthy donor with previous COVID-19; O-CoV, oncologic patient with previous COVID-19; O-V, vaccinated oncologic patients; O-CoV-V, vaccinated oncolgic patients with previous COVID-19; *, **, *** and **** indicate significant ($p<0.05$) , very significant ($p<0.01$) and highly significant ($p<0.001$) differences, respectively.

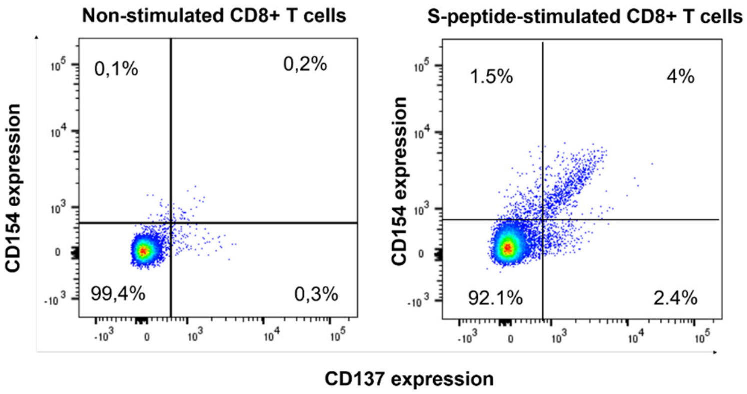

Figure S6. Flow cytometry and gating strategy for quantification of activated CD8 T cells. Representative flow cytometry density plots of CD154 and CD137 co-expression profiles in CD8 T cells from donors before and after stimulation with S-peptides, as indicated.

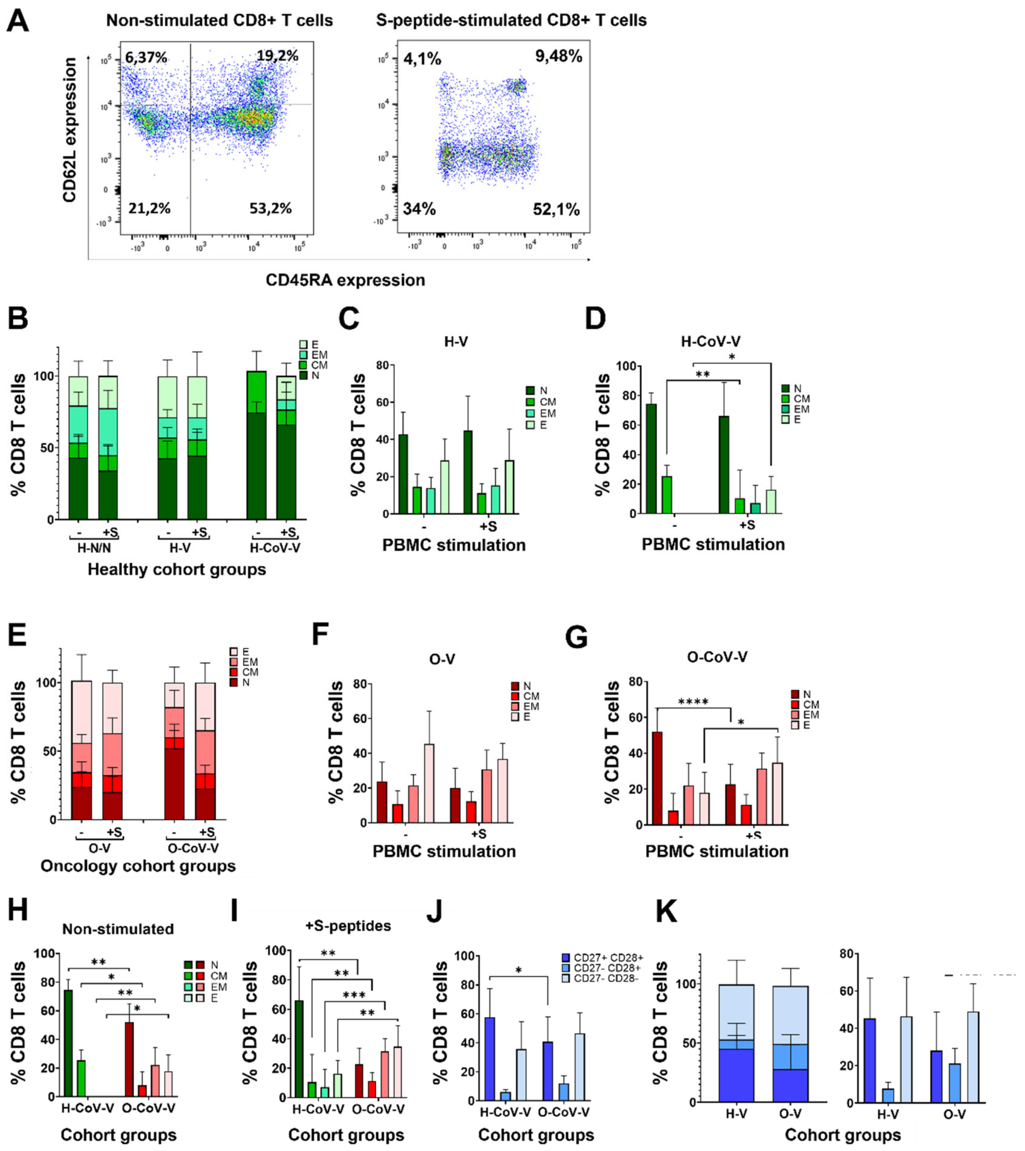

Figure S7. Differentiation phenotypes in CD8 T cells. **(A)** Representative flow cytometry density plots with CD62L-CD45RA co-expression profiles in CD8 T cells before and after the stimulation with S-peptides. Quadrants were established with unstained controls. Percentages of the corresponding populations are shown within the quadrants. **(B, C, D)** Relative percentages of CD8 T cell differentiation phenotypes from the indicated healthy donors and oncologic patient. Means and error bars (standard deviations) are shown. N, CM, EM and E, indicate naïve-stem cell (CD62L+ CD45RA+), central memory (CD62L+ CD45RA-), effector memory (CD62L- CD45RA-) and effector (CD62L- CD45RA+) phenotypes. **(E, F, G)** Relative percentages of CD8 T cell differentiation phenotypes from the indicated healthy donors and oncologic patients. **(H, I)** Relative percentages of CD8 T cell differentiation phenotypes in H-CoV-V and O-CoV-V groups before and after stimulation with S-peptides. **(J, K)** Relative percentages of CD8 T cell differentiation phenotypes in V-H-CoV, V-O-CoV, H-V and O-V groups as indicated in the graphs. CD27+ CD28+, CD27- CD28+ and CD27+ CD28+ indicate poorly differentiated, intermediate differentiated and highly differentiated T cell phenotypes. **(B-K)** Statistical significance was tested by ANOVA followed by Tukey´s pair-wise comparisons. H-N/N, non-vaccinated, non-COVID-19 donors; H-CoV, healthy donors with previous COVID-19 infection; H-V, vaccinated healthy donor; H-CoV-V, vaccinated healthy donor with previous COVID-19; O-CoV, oncologic patient with previous COVID-19; O-V, vaccinated oncologic

patients; O-CoV-V, vaccinated oncolgic patients with previous COVID-19; *, **, *** and **** indicate significant (p<0.05) , very significant (p<0.01) and highly significant (p<0.001) differences, respectively.

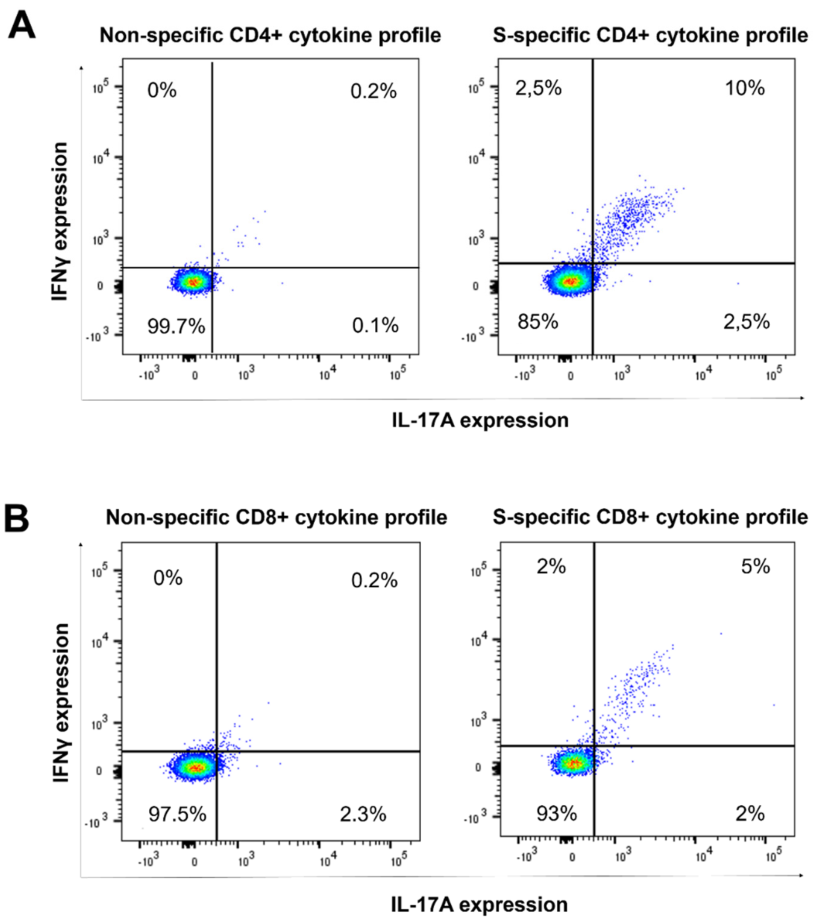

Figure S8. Flow cytometry and gating strategy for quantification of cytokine expression within activated T cells. **(A, B)** Representative flow cytometry density plots with the expression of INFγ and IL-17 before and after stimulation with S peptides in CD4 T cells **(A)** and in CD8 T cells **(B)**.
